# Pregnancy Rate after Myomectomy and Associated Factors among Reproductive Age Women Who Had Myomectomy at Saint Paul's Hospital Millennium Medical College, Addis Ababa: Retrospective Cross-Sectional Study

**DOI:** 10.1155/2021/6680112

**Published:** 2021-11-28

**Authors:** Meseret Jeldu, Tadios Asres, Temesgen Arusi, Muluken Gunta Gutulo

**Affiliations:** ^1^Wolkite University, Ethiopia; ^2^Saint Paul's Millennium Medical College, Ethiopia; ^3^Wolaita sodo univesrsity, Ethiopia

## Abstract

**Introduction:**

Uterine myoma occurs in 20-50% of reproductive age women. Uterine myomas may be associated with 5-10% of cases of infertility, but it is the sole cause or factor in only 2-3% of all infertility cases. Myomectomy is surgery done to remove myoma regardless of the methods.

**Objective:**

To assess impact of myomectomy on pregnancy rate and associated factors among reproductive age women who had myomectomy at St. Paul's Hospital Millennium Medical College, in Addis Ababa. *Methodology*. Hospital-based retrospective cross-sectional study was conducted to determine pregnancy rate after myomectomy and its associated factors. Patients who had myomectomy in SPHMMC from September 2012 to September 2017 were enrolled. Information was retrieved from hospital records and phone interviews with the patients. The strength of statistical association was measured by adjusted odds ratios and 95% confidence intervals. Statistical significance was declared at *p* value < 0.05.

**Result:**

Among 180 females participated in this study, 52.2% got pregnant after myomectomy. The result showed that females with age > 35 years were 0.31 times less likely to get pregnant after surgery than those ages 20-25 years [AOR = 0.31 (95% CI: 0.29-0.54)]. People with no infertility before surgery were 1.19 times more likely to be pregnant after surgery than those with unexplained infertility before the surgery [AOR = 1.19 (95% CI: 1.06-1.57)]. People with two uterine incisions were 0.06 times less likely [AOR = 0.06 (95% CI: 0.043-0.51)] while those with three or more than three incisions were 0.02 times less likely [AOR = 0.02 (95% CI: 0.002-0.22)] to get pregnant compared with those with one incision on uterine wall.

**Conclusion:**

Age, number of incision, and infertility before surgery were significantly associated with rate of pregnancy after myomectomy.

## 1. Introduction

Uterine myoma is a monoclonal, benign tumor arising from smooth muscles of the uterus. It occurs in 20-50% of reproductive age women and can be identified by ultrasound in approximately 80% of African-American and 70% of white American women by the time they reach menopause [1–3]. These tumors are classified based on their location and direction of growth: submucous, intramural, and subserous. Submucous myomas are proximate to the endometrium and grow toward and bulge into the endometrial cavity [1, 4–6].

The role of myomas as a causative factor in infertility remains controversial. It has been proposed that myomas interfere sperm transport, cause cervical and/or tubal blockage, distort endometrial cavity, or may cause inflammation to endometrium or secretion of vasoactive substances [2]. According to AFS guideline for practice, myomas may be associated with 5 to 10% of infertility but it is a sole factor in only 2 to 3% of all infertility cases. Even though myomectomy is the recommended treatment for symptomatic myomas among women wishing to preserve their fertility, the fertility performance of the women after this surgery is not thoroughly studied and detailed. It may also be associated with infertility due to extensive adhesion causing tubal blockage and intrauterine adhesions (IUAs) if endometrium is breached during surgery [1–3].

Myomectomy is found to significantly increase conception rate in women with myomas as a sole factor of infertility. The conception rate following myomectomy ranged from 25 to 77% in available retrospective studies [3]. In different retrospective study done at different hospitals and country showed improvement in fertility after myomectomy. Moreover, age of the women, presence of pelvic adhesion, and the presence of concomitant history of unexplained infertility have negative influence on chance of conception after myomectomy. There was no significant difference in pregnancy rate according to whether the uterine cavity is entered or not, preoperative size, location, and size of myoma(s) [2, 3, 7–9].

Many of the women with symptomatic myoma are in reproductive age group and need for future fertility [2, 7, 10–12]. There is shortage of data done in this area of interest as well as there is no research done on fertility performance after myomectomy in Ethiopia. For this reason and due to uncertainty of effect of myomectomy on pregnancy rate, physicians always face dilemma in counseling and management options of women with symptomatic myomas who need to conserve their fertility.

## 2. Materials and Methods

### 2.1. Study Area, Design, and Populations

Institution-based cross-sectional study was undertaken at St. Paul's Hospital Millennium Medical College, in Addis Ababa, the capital city of Ethiopia, from November 1, 2018, to December 30, 2018. It is the hospital with highest delivery rate and gives care to most of gynecologic patients in the city and other parts of the country. All women who had myomectomy at St. Paul's Hospital Millennium Medical College from September 2012 to September 2017 who are eligible were included under the study.

### 2.2. Sample Size Determination and Sampling Technique

All women who had abdominal myomectomy from September 2012 to September 2017 were included.

### 2.3. Inclusion and Exclusion Criteria

#### 2.3.1. Inclusion Criteria

Women aged from 15 to 40 years of age at the time of surgery, those who had abdominal myomectomy from September 2012 to September 2017 and who volunteer to give information on phone interview.

#### 2.3.2. Exclusion Criteria

Women with other significant infertility factor (i.e., tubal factor and abnormal SA of partner), those who are not wishing to conceive after myomectomy, those who had bilateral tubal ligation (BTL), those who have no phone number on patient medical record, and those who are not responding to phone call are unmatching identity between medical record name and phone responder.

### 2.4. Data Collection

The patient identification number of all cases of women who had abdominal myomectomy was taken from the hospital register of patient admission, operation room log book, and discharge log book from department of OBGYN. Their case files were retrieved from the medical record department of the hospital. A structured questionnaire written in English and customized for this study was used for data collection from each patient chart. The questionnaire includes sociodemographic, indication of myomectomy, intraoperative findings, and surgical techniques. Additionally, phone call was made to each patient for additional data that is not recorded in patient chart. Data collectors or interviewers were three trained OBGYN residents. The data collectors were trained for one day about the objectives of the study, techniques of collection, and how to fill the questionnaire. Every day, all of the collected data was reviewed and checked for completeness and relevance by the principal investigator. The collected data was cleaned and entered before analysis. The participants were informed that confidentiality of the information collected and the test result was insured.

### 2.5. Data Analysis

Data were checked for completeness and entered into Epi Info version 7.1 and then exported to SPSS version 23 for further data cleaning and analysis. Frequency distributions were obtained to check for data entry error (missing/unrecognized values and codes). Descriptive statistics, tables, graphs, means, and frequency distribution were used to present the information. The presence of an association between the independent and outcome variable was checked by the Pearson chi-square test. Additionally, each independent variable was fitted separately into bivariate logistic analysis to evaluate for the degree of association with the rate of pregnancy. Also, a further degree of association was assessed by multivariate logistic regression on variables with *p* values less than 0.25. The significance level was obtained with 95% CI and *p* value < 0.05 to evaluate the degree of association between factors and the rate of pregnancy.

#### 2.5.1. Operation Definitions

Abortion is the process of pregnancy termination before the gestational age of viability (28 weeks/07months).

Spontaneous abortion is an abortion without medical or surgical intervention.

Abnormal uterine bleeding (AUB) is defined as menstrual bleeding that is not regular and cyclic and normal.

Infertility is one year unprotected sexual intercourse without conception.

Unexplained infertility is infertility in which there is no major causative factor in both partners.

Recurrent abortion refers to two or more consecutive spontaneous abortions.

## 3. Results

### 3.1. Socio Demographic Characteristics

A total of 180 women were included in this study. The mean (±SD) age of the women was 30 (±5.4 SD) and ranged from 20 to 40 years. The age distribution of the participant showed that large proportion 69 (38.3%) belongs to the age group of 26-30. The distribution of the respondent by educational status revealed that 68 (37.8%) of women had no formal education. The majority of the mothers were 62 (36.5%) housewives. More than half of the participant 99 (55.5%) of the women were urban residents (Table 1).

### 3.2. Clinical Characteristics of Participants before Myomectomy

AUB was the primary indication for myomectomy for 80 (44.4%) of women, and for 42 (23.3%), pelvic pain was the primary indication for myomectomy. Unexplained infertility was the primary indication for myomectomy for 36 (20%) of the women. Half of the women 90 (50%) had infertility before surgery. One hundred fourteen (63.3%) of the women were nulligravid whereas 120 (66.7%) of the women were nulliparous. Regarding uterine size before surgery, 161 (89.4%) of the participants had ≥12 weeks uterine size (Table 2).

### 3.3. Intraoperative Finding of the Study Participants

Among females participated in this study, 75 (41.7%) of them had 1 to 3 myomas removed. For one hundred forty-seven (81.7%) of them, the largest size of myoma removed was >5 cm. Regarding location of the largest myoma removed, 136 (75.9%) of the participants belong to intramural type. In eighty-seven (48.3%) women, myomectomy was done through one incision on uterine wall. For 80 (44.4%) of the participants, location of incisions was on both posterior and anterior wall of the uterus. Fifty-six (31.1%) of the participant had endometrial cavity entered while undergoing the surgery (Table 3).

### 3.4. Pregnancy Rate after Myomectomy

**Figure 1:**
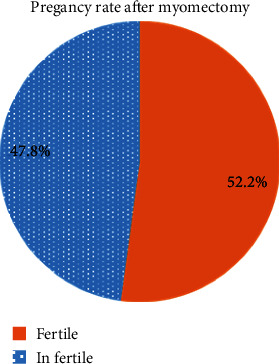
Pregnancy after myomectomy.

Out of the 180 female who underwent myomectomy, 94 (52.2%) of them became pregnant after myomectomy while 86 (47.8%) of them did not get pregnant after myomectomy. Half of the participants were diagnosed with unexplained infertility before the surgery. The pregnancy rate was found to be 56.7% after myomectomy. Of thirty-six patients for whom myomectomy was done for unexplained infertility as a primary indication, 20 (55.6%) became pregnant (Figure 1).

### 3.5. Factors Associated with Pregnancy Rate after Myomectomy

Bivariate analysis was performed, and taking *p* value < 0.25 as a cut of point for determining significance, then multivariate analysis was conducted for those with *p* value < 0. 25. The seven covariates that were significantly associated (*p* value < 0.25) with fertility after myomectomy at the bivariate analysis were fitted into the backward stepwise multivariable logistic regression model to control for confounding. The final predictors of pregnancy rate after myomectomy were age, infertility before surgery, and number of incision (*p* value < 0.05). Age was significantly associated with pregnancy rate after myomectomy. Participants aged >35 years were 0.31 times less likely to get pregnant after surgery than those aged 20-25 years [AOR = 0.31 (95% CI: 0.29-0.54)]. Infertility before surgery was the other predictor. People with no infertility before surgery were 1.19 times more likely to be pregnant after surgery than those with unexplained infertility before the surgery [AOR = 1.19 (95% CI: 1.06-1.57)]. Number of incision was also statistically significant in that people with two incisions were 0.06 times less likely while those with ≥3 incisions were 0.02 times less likely to get pregnant than those with one incision on the uterine wall [AOR = 0.06 (95% CI:0.05-0.51)] and [AOR = 0.02 (95% CI: 0.002-0.22)], respectively (Table 4).

## 4. Discussion

This is a health facility-based retrospective cross-sectional study aimed to assess the magnitude of pregnancy rate after myomectomy and associated factor(s) among women who underwent abdominal myomectomy surgery in SPHMMC from September 2012 to September 2017. The finding revealed that magnitude of fertility after myomectomy was significantly associated with age, number of incisions, location of incision, and infertility before surgery.

Among female participated in this study 52.2% of the females got pregnant after myomectomy. This is almost similar to a study done in China which found that 50.3% of women after myomectomy were able to conceive [13]. The result of this study was higher than a study done in Hungary which stated that 40.4% of women got pregnant after myomectomy [14]. On the other hand, our study finding was lower than a study done in the UK which found that 57% pregnancy rate after myomectomy [3]; the finding from the USA found the same result as the UK finding which 57% pregnancy rate was after surgery [15]. Similar study done in New York also found 65.2% of pregnancy rate [16]. But in this study, there are 43 (23.9%) females whose history of infertility before surgery is unknown so that the overall prevalence of pregnancy after myomectomy may be affected negatively.

This study found that age was significantly associated with pregnancy rate after myomectomy. Participants aged >35 years were less likely to be pregnant than those age group 20-25 years. In a study done at Jessop Hospital, UK, women aged ≤35 had a significantly higher chance of conception (74%) than women ≥36 years (30%) [3]. Other study done in China also found that younger people have a higher pregnancy rate than older [13]. A study done in Senegal also support the result of this study; younger people get pregnant than those older people after myomectomy [17].

Number of incisions was also significantly associated with pregnancy rate after myomectomy. Participants who had both two and three and more than three incisions were less likely to get pregnant after myomectomy than those with only one incision. This can be explained by that as the number of incisions increases, the risk of adhesion possibly blocking fallopian tubes also increases [3, 7, 8]. Entry into endometrium cavity occurred on 56 females (45.16%) and has no association with the rate of pregnancy after the myomectomy. This finding is supported by other studies done in China and Hungary [13, 14].

Infertility before surgery was also significantly associated with pregnancy rate after myomectomy. Participants who had a known infertility were less likely to get pregnant after myomectomy than those who did not complain failure to conceive before surgery. Infertility before myomectomy is a factor of poor prognosis for the subsequent pregnancy [17].

Although the overall pregnancy rate is relatively lower (52.2%) because of inclusion of patients whose fertility history before surgery was not known, Myomectomy improved pregnancy rate by 55.7% in patients with unexplained infertility. This figure is higher than the study done at Johns Hopkins University of school of medicine (51%) and lower than the one done at North Shore University Hospital (65.2%) [15, 16].

## 5. Limitation of the Study

Since this is retrospective study, we cannot reach to cause and effect associations. Additionally, this study was done in those patients for whom open laparotomy was done for myomectomy which is almost obsolete in other sites (endoscopic procedure) as result difficult to get data on myomectomy done with open laparotomy, because of lack of investigative modalities to properly put myoma as per FIGO classification, which is very important to correctly identify the location of myoma relative of endometrium and tubes.

## 6. Conclusion

Overall, half of the participants were able to get pregnant after myomectomy. Age, number of incisions, infertility before surgery, and location of incision were significantly associated with rate of pregnancy after myomectomy. Myomectomy improved pregnancy rate in those who had unexplained infertility before the index surgery.

## Figures and Tables

**Table 1:** Sociodemographic characteristics of women who underwent myomectomy in SPHMMC, Addis Ababa, 2019.

Variable	Frequency
Age	
20-25	33
26-30	69
31-35	49
>35	29
Place of residence	
Urban	99
Rural	81
Education	
No formal education	68
Primary school	19
Secondary school	31
College	43
Unregistered	19
Occupation	
Housewife	62
Government employee	48
Merchant	21
Farmers	28
Private	21

**Table 2:** Clinical characteristics before myomectomy of women who underwent myomectomy in SPHMMC, Addis Ababa, 2019.

Variable	Frequency
Primary indication for myomectomy	
AUB	80
Recurrent abortion	5
Unexplained infertility	36
Pelvic pain	42
Other	17
Infertility before surgery	
Yes	90
No	47
Unknown	43
Gravidity	
0	114
1	27
2	28
≥3	11
Parity	
0	120
1	31
2	23
≥3	6
Abortion before surgery	
Yes	27
No	153
Uterine size before surgery	
<12	19
≥12	161

**Table 3:** Intraoperative finding of women who underwent myomectomy, SPHMMC, Addis Ababa, 2019.

Variable	Frequency	Percentage
Number of myomas removed		
1-3	75	41.7
4-5	39	21.7
>5	60	33.3
Unspecified	6	3.3
Size of the largest myoma removed (cm)		
≤5	21	11.7
>5	147	81.7
Unspecified	12	6.7
Location of the largest myoma removed		
Submucous	24	13.4
Intramural	136	75.9
Subserous	13	7.3
Unspecified	6	3.4
Number of incision		
1	87	48.3
2	53	29.4
≥3	16	8.9
Unspecified	24	13.3
Location of incision(s) on the uterus		
Anterior wall	67	37.2
Posterior wall	21	11.6
Both anterior and posterior wall	80	44.4
Unspecified	12	6.7
Endometrial cavity entered		
Yes	56	31.1
No	124	68.9

**Table 4:** Bivariate and multivariate logistic regression analysis of factors associated with fertility after myomectomy, SPHMMC, Addis Ababa, 2019.

Variable	Fertility after myomectomy		COR (95% CI)	AOR (95% CI)
	Yes, *n* (%)	No, *n* (%)		
Age				
20-25	13 (39.4)	20 (60.6)	1	
26-30	40 (57.9)	29 (42.0)	0.47 (0.20-1.09)	0.81 (0.27-2.46)
31-35	27 (55.1)	22 (44.9)	0.52 (0.21-1.29)	1.29 (0.35-4.69)
>35	14 (48.3)	15 (51.7)	0.69 (0.25-1.91)	0.31 (0.29-0.54)^∗^
Primary indication for myomectomy				
AUB	42 (52.5)	38 (47.5)	1	
Recurrent abortion	4 (80.0)	1 (20.0)	0.28 (0.03-2.58)	0.21 (0.01-4.23)
Unexplained infertility	20 (55.6)	16 (44.4)	0.88 (0.40-1.94)	1.67 (0.50-5.48)
Pelvic pain	22 (52.4)	20 (47.6)	1.01 (0.47-2.12)	1.25 (0.45-3.46)
Other	6 (35.3)	11 (64.7)	2.02 (0.68-6.01)	1.65 (0.35-7.69)
Infertility before surgery				
Yes	51 (56.7)	39 (43.3)	0.41 (0.19-0.86)	0.30 (0.10-0.89)
No	28 (59.6)	19 (40.4)	1.36 (0.15-0.85)	1.19 (1.06-1.57)^∗^
Unknown	15 (34.9)	28 (65.1)	1	
Uterine size before surgery				
<12	13 (68.4)	6 (31.6)	1	
≥12	81 (50.3)	80 (49.7)	2.13 (0.77-5.91)	1.51 (0.48-4.27)
No. of myomas removed				
1-3	36 (48.0)	39 (52.0)	1	
4-5	24 (61.5)	15 (38.5)	0.57 (0.26-1.27)	0.92 (0.33-2.57)
>5	33 (55.0)	27 (45.0)	0.75 (0.38-1.49)	1.34 (0.47-3.79)
Unspecified	—	4 (100.0)	—	—
No. of incision				
1	37 (42.5)	50 (57.5)	1	
2	33 (62.3)	20 (37.7)	0.45 (0.22-0.90)	0.06 (0.05-0.51)^∗^
≥3	11 (68.8)	5 (31.3)	0.33 (0.10-1.05)	0.02 (0.002-0.22)^∗^
Unspecified	13 (54.2)	11 (45.8)	0.62 (0.25-1.55)	0.08 (0.01-0.62)
Location of incision on the uterus				
Anterior wall	33 (49.3)	34 (50.7)	1	
Posterior wall	8 (38.1)	13 (61.9)	1.58 (0.58-4.29)	2.70 (0.73-9.95)
Both anterior and posterior wall	48 (60.0)	32 (40.0)	1.64 (0.33-1.94)	3.09 (0.98-51.04)
Unspecified	5 (41.7)	7 (58.3)	1.35 (0.39-4.71)	5.69 (0.57-11.64)

^∗^ *p* value < 0.05.

## Data Availability

Data are available on request from the corresponding author.

## Abbreviations

AFS: American Fertility Society
BS: Bilateral salpingectomy
BTL: Bilateral tubal ligation
HSG: Hysterosalpingography
IUA: Intrauterine adhesion
OBGYN: Obstetrics and Gynecology
RPL: Recurrent pregnancy loss
TAH: Tikur Anbessa Hospital
SA: Semen analysis
SPHMMC: Saint Paul's Hospital Millennium Medical College
